# Supplementary material for: SSnet: A Deep Learning Approach for Protein-Ligand Interaction Prediction
Source: Int J Mol Sci. 2021 Jan 30;22(3):1392. doi: 10.3390/ijms22031392 (PMC7869013; doi:10.3390/ijms22031392)
Supplement: Supplementary file 1 [file ijms-22-01392-s001.pdf]

Predicting Potential SARS-COV-2 Drugs - In Depth Drug Database Screening Using Deep Neural Network Framework SSnet, Classical Virtual Screening and Docking

Nischal Komal Karki,[†,‡] Niraj Verma,[†,‡] Francesco Trozzi,[†,‡] Peng Tao,[†] Elfi Kraka,[†] and Brian Zoltowski[*,†]

†*Department of Chemistry, Southern Methodist University, Dallas TX USA*

‡*Authors contributed equally*

E-mail: bzoltowski@mail.smu.edu

Timing and resources for SSnet

The SSnet model was tested on SMU Maneframe II. The GPU node is equiped with 36 accelerator nodes with NVIDIA GPUs, dual Intel Xeon E5-2695v4 2.1 GHz 18-core "Broadwell" processors, 256 GB of DDR4-2400 memory, and one NVIDIA P100 GPU accelerator. Each NVIDIA P100 GPU has 3,584 CUDA cores and 16 GB CoWoS HBM2 memory. The P100 GPU is based on the new Pascal architecture and an extremely high bandwidth (732 GB/s) stacked memory architecture. It took around 36 minutes to screen through 1 million compounds per target including the preprocessing of the data (featurization of ligands and proteins).

Introduction to Hilbert Space Filling Curve

Space filling curves are a class of curves in mathematical analysis that are used to map every point of a 1-dimensional line into every ordered-pair in 2-dimensional space. Hilbert's space filling curve is an example of such a curve. The curve is defined recursively such that with each recursion the line occupies more space, ultimately filling it after infinite recursions. A first order Hilbert curve divides a given space into quadrants whose center points are joined consecutively without repeating or intersecting the curve (similarly to the classic snake game). A second order curve further subdivides the quadrants whose centers become new points. The points are first connected within the sub-quadrants and the sub-quadrants are rotated such that the end-points of each sub-quadrant can be connected to its neighbor. This process is recursively done until the line spans the entire 2D space. In the present study, we recur this algorithm until the number of ordered-pair generated is less than or equal to the number of compounds to be mapped. A python script named plot_hill.py can be found in https://github.com/nischal-karki/chem-hilbert-web-host/, this script was used to generate the required dimension of 2D image for each of the datasets.

SSnet and smina scores

Top scores for both smina and SSnet

Table S1: Top Scores for SSnet and smina

Drug name	smina ACE2 (open)	smina ACE2:S1 (closed)	smina ACE2 (closed)	SSnet ACE2 (open)	SSnet ACE2:S1 (closed)	SSnet ACE2 (closed)	Common name	Usage	Mode of Action
ZINC000100378061	-10.23 ± 0.62	-10.83 ± 0.38	-10.00 ± 0.93	0.999	0.999	0.999	Naldemedine	Opiod induced constipation	Opiod receptors
ZINC000003978005	-10.23 ± 0.44	-10.13 ± 0.58	-10.40 ± 0.40	0.983	0.963	0.983	Dihydroergotamine	Treatment of migraine disorders	Serotonine, dopamine and adrenoreceptor antagonist (RAAS related)
ZINC000077313075	-9.9 ± 0.27	-10.16 ± 0.36	-9.93 ± 0.51	0.984	0.967	0.985	Sorafenib Beta-D-Glucuronide	Cancer	Kinase inhibitor
DB11986	-10.1 ± 0.67	-10.30 ± 0.53	-9.90 ± 0.80	0.978	0.954	0.979	Entrectinib	Treatment of non-small cell lung cancer	Tropomyosin receptor tyrosine (TRK), proto-oncogene tyrosine-protein kinase ROS1, kinase and anaplastic lymphoma kinase (ALK) inhibitor.
ZINC000001612996	-9.53 ± 0.29	-9.50 ± 0.60	-9.87 ± 0.36	0.976	0.949	0.977	Irinotecan	Treatment for colorectal cancer	Antineoplastic enzyme inhibitor
ZINC000043195321	-9.33 ± 0.42	-9.90 ± 0.00	-9.10 ± 0.53	0.964	0.926	0.966	Capmatinib	Treatment of non-small cell lung cancer	Tyrosine kinase Inhibitor
ZINC000003781738	-9.53 ± 0.44	-9.47 ± 0.62	-9.73 ± 0.44	0.967	0.931	0.968	Lestaurtinib	Cancer	Tyrosine kinase inhibitor
ZINC000205224698	-9.07 ± 0.51	-9.20 ± 0.60	-9.43 ± 0.04	0.993	0.985	0.993	Fosnetupitant	Prevention of chemotherapy-induced nausea and vomiting	Neurokinin 1 (NK1) receptor antagonist
DB12329	-9.13 ± 0.38	-9.43 ± 0.38	-9.07 ± 0.62	0.992	0.983	0.992	Eravacycline	Antibiotic	β-lactamase inhibitors
ZINC000006716957	-10.10 ± 0.53	-10.57 ± 0.04	-9.73 ± 0.58	0.784	0.628	0.788	Nilotinib	Treatment for chronic myelogenous leukemia	Bcr-Abl tyrosine kinase inhibitor
DB15035	-9.03 ± 0.42	-9.30 ± 0.27	-8.83 ± 0.58	0.998	0.995	0.998	Zanubrutinib	Treatment of mantle cell lymphoma	Tyrosine kinase inhibitor
ZINC000022448696	-9.16 ± 0.08	-9.17 ± 0.09	-9.3 ± 0.00	0.998	0.995	0.998	Indinavir	Anti-viral	HIV protease inhibitor

SSnet scores are probability, in the range from 0 to 1, of a drug binding with a IC50 less then 10nM. Smina values are provided in kcal/mol. Values represent mean of 3 replicas. The deviation reported is calculated using the average of absolute deviations from the mean values of the 3 smina replicas.

Top scores with SSnet

Table S2: Top Scorers for SSnet

Drug name	smina ACE2 (open)	smina ACE2:S1 (closed)	smina ACE2 (closed)	SSnet ACE2 (open)	SSnet ACE2:S1 (closed)	SSnet ACE2 (closed)	Common name	Usage	Mode of Action
ZINC000095551509	-8.80 ± 0.20	-8.97 ± 0.36	-8.87 ± 0.36	0.998	0.999	0.999	Grazoprevir	Treatment of hepatitis C	Inhibitor of NS3/4A serine protease
DB09078	-7.83 ± 0.18	-7.90 ± 0.13	-7.80 ± 0.13	0.998	0.999	0.999	Lenvatinib	Treatment of thyroid cancer	Tyrosine kinase (RTK) inhibitor
DB14723	-8.37 ± 0.44	-8.80 ± 0.13	-8.00 ± 0.13	0.997	0.999	0.999	Larotrectinib	Cancer	Tropomyosin receptor kinase (Trk) inhibito
ZINC000011677857	-8.57 ± 0.16	-8.73 ± 0.38	-8.67 ± 0.38	0.997	0.999	0.999	Avanafil	Erectile dysfunction	Phosphodiesterase-5 inhibitor
DB11581	-9.77 ± 0.38	-9.93 ± 0.49	-10.23 ± 0.49	0.997	0.998	0.999	Venetoclax	Treatment chronic lymphocytic leukemia	BCL-2 inhibitor
DB08916	-7.70 ± 0.20	-7.70 ± 0.27	-7.77 ± 0.27	0.997	0.998	0.998	Afatinib	Treatment of metastatic non-small cell lung cancer	Tyrosine kinase inhibitor
DB08890	-8.23 ± 1.78	-7.97 ± 1.36	-7.87± 1.36	0.996	0.998	0.998	Linaclotide	Treatment of irritable bowel syndrome	Guanylate cyclase 2C agonist
ZINC000001319780	-8.17 ± 0.56	-8.40 ± 0.53	-8.07 ± 0.53	0.996	0.998	0.998	Buprenorphine	Treatment of severe pain	Partial mu-opioid receptor agonist
DB13879	-9.97 ± 0.49	-9.80 ± 0.47	-10.03 ± 0.47	0.995	0.998	0.998	Glecaprevir	Treatment of hepatitis C	Inhibitor of NS3/4A serine protease
ZINC000218037687	-9.37 ± 0.42	-9.5 ± 0.87	-9.73 ± 0.87	0.994	0.997	0.997	Voxilaprevir	Treatment of Hepatitis C	Inhibitor of NS3/4A serine protease
DB00834	-8.23 +-/ 0.31	-8.27 ± 0.36	-8.27 ± 0.36	0.993	0.997	0.997	Mifepristone	Treatment of hypercortisolism	Glucocorticoid receptor antagonist
ZINC000004393164	-6.77 ± 0.18	-6.80 ± 0.27	-6.47 ± 0.27	0.993	0.997	0.997	Aliskiren	Hypertension	Renin inhibitor (Part of RAAS)

SSnet scores are probability, in the range from 0 to 1, of a drug binding with a IC50 less then 10nM. Smina values are provided in kcal/mol. Values represent mean of 3 replicas. The deviation reported is calculated using the average of absolute deviations from the mean values of the 3 smina replicas.

Top Scores for SSnet ACE2 (open) - ACE2:S1 (closed)

Table S3: Top Scores for SSnet ACE2 (open) - ACE2:S1 (closed)

Drug name	smina ACE2 (open)	smina ACE2:S1 (closed)	smina ACE2 (closed)	SSnet ACE2 (open)	SSnet ACE2:S1 (closed)	SSnet ACE2 (closed)	Common name	Usage	Mode of Action
DB13125	-7.00 ± 0.20	-7.60 ± 0.73	-7.23 ± 0.98	0.629	0.442	0.633	Lusutrombopag	Treatment of thrombocytopenia	Thrombopoietin receptor (TPOR) agonist
DB00301	-8.00 ± 0.47	-7.97 ± 0.62	-8.17 ± 0.49	0.607	0.423	0.611	Flucloxacillin	Antibiotic	Beta-lactamase inhibitor
ZINC000030731319	-9.30 ± 0.27	-9.30 ± 0.40	-9.43 ± 0.31	0.634	0.451	0.638	Morphine-3-glucuronide	Metabolite of morphine	Mu and kappa opioid receptors agonist
ZINC13682481	-7.87 ± 0.24	-8.00 ± 0.13	-7.73 ± 0.31	0.637	0.454	0.641	Elvitegravir	Treatment of HIV infection	HIV-1 integrase strand transfer inhibitor
ZINC100054334	-8.37 ± 0.11	-8.17 ± 0.11	-8.30 ± 0.2	0.629	0.448	0.633	p-O-glucuronide rosiglitazone	Metabolite of rosiglitazone (Anti-diabetic)	PPAR receptors binder
ZINC000005116499	-8.40 ± 0.47	-8.70 ± 0.027	-8.13 ± 0.64	0.627	0.446	0.632	4-hydroxymidazolam	Metabolite of midazolam (treatment of actute seizures)	Gamma-aminobutyric acid (GABA) inhibitor
ZINC000004744090	-8.13 ± 0.51	-8.03 ± 0.58	-8.37 ± 0.35	0.692	0.512	0.697	Methylprednisone	Treatment of Leukemia	Steroid. Not available
DB08882	-8.63 ± 0.38	-8.80 ± 0.33	-8.53 ± 0.51	0.742	0.563	0.747	Linagliptin	Treatment of type II diabetes	DPP-4 inhibitor
ZINC000003882897	-8.57 ± 0.44	-8.97 ± 0.22	-8.37 ± 0.62	0.699	0.522	0.704	Pyronaridine	Treatment of Malaria	Not available
ZINC000043205655	-8.63 ± 0.64	-8.97 ± 0.49	-8.33 ± 0.91	0.653	0.475	0.659	Telotristat ethyl	Treatment of carcinoid syndrome diarrhea	Serotonin synthesis inhibitor
ZINC5843546	-7.20 ± 0.27	-7.57 ± 0.04	-7.07 ± 0.29	0.735	0.559	0.741	Azosemide	Hypertension	Not available. Potentially interact with RAAS
ZINC5104028	-8.03 ± 0.38	-8.03 ± 0.18	-7.83 ± 0.31	0.653	0.478	0.657	Ormeloxifene	Oral contraceptive, anti-cancer	Non-steroidal Selective Estrogen Receptor Modulator[e]

SSnet scores are probability, in the range from 0 to 1, of a drug binding with a IC50 less then 10nM. Smina values are provided in kcal/mol. Values represent mean of 3 replicas. The deviation reported is calculated using the average of absolute deviations from the mean values of the 3 smina replicas.

SSnet scores with and without Zinc of known RAAS interacting molecules

Table S4 shows the difference in SSnet scores for known RAAS interacting molecules in presence and absence of zinc. This table provides an indication of the positive influence of Zinc cation in the binding probabilities of these compounds, suggesting a positive cooperation of Zinc and these molecules in binding to the ACE2 receptor.

Table S4: SSnet scores with and without Zinc of known RAAS interacting molecules.

	6M1D-SSnet-Zn	6M17-SSnet-Zn	6M18-SSnet-Zn	6M1D-SSnet	6M17-SSnet	6M18-SSnet	Common name	Function
DB00722	0.855	0.715	0.857	0.804	0.662	0.809	Lisinopril	ACE Inhibitor
DB01197	0.155	0.150	0.153	0.127	0.135	0.125	Captopril	ACE Inhibitor
DB00542	0.208	0.185	0.205	0.167	0.162	0.165	Benazepril	ACE Inhibitor
DB01340	0.693	0.532	0.692	0.627	0.503	0.629	Cilazapril	ACE Inhibitor
DB09477	0.779	0.614	0.781	0.686	0.552	0.688	Enalaprilat	ACE Inhibitor
ZINC000003801163	0.842	0.706	0.844	0.799	0.672	0.803	Quinapril	ACE Inhibitor
ZINC000003875259	0.659	0.529	0.658	0.529	0.442	0.529	Valsartan	ACE2 receptor blocker
DB00275	0.274	0.216	0.271	0.140	0.142	0.138	Olmesartan	ACE2 receptor blocker
DB00876	0.286	0.253	0.282	0.147	0.159	0.142	Eprosartan	ACE2 receptor blocker
DB00966	0.363	0.273	0.360	0.101	0.112	0.098	Telmisartan	ACE2 receptor blocker
DB01029	0.902	0.800	0.903	0.870	0.763	0.874	Irbesartan	ACE2 receptor blocker
ZINC000004074875	0.903	0.806	0.905	0.809	0.689	0.813	Candesartan	ACE2 receptor blocker

SSnet scores are probability, in the range from 0 to 1, of a drug binding with a IC50 less then 10nM. Smina values are provided in kcal/mol. Values represent mean of 3 replicas. The deviation reported is calculated using the average of absolute deviations from the mean values of the 3 smina replicas.

SSnet scores with and without Zinc on top SSnet scores

Table S5: Zinc effect on SSnet binding probabilities

	6M1D-SSnet-Zn	6M17-SSnet-Zn	6M18-SSnet-Zn	6M1D-SSnet	6M17-SSnet	6M18-SSnet	Common name	Usage
DB01180	0.761	0.637	0.759	0.462	0.371	0.459	Rescinnamine	ACE Inhibitor
ZINC000261494566	0.791	0.712	0.789	0.508	0.416	0.504	Rifabutin	Macrolide
DB01129	0.769	0.592	0.772	0.436	0.349	0.435	Rapeprazole	Proton Pump inhibitor
ZINC000008577218	0.627	0.470	0.628	0.216	0.216	0.213	Folic Acid	Vitamin B9
DB01228	0.787	0.626	0.789	0.485	0.384	0.484	Encainide	Sodium channel blocker
ZINC000021981290	0.695	0.513	0.696	0.327	0.274	0.326	5'-O-Desmethyl Omeprazole	Proton Pump inhibitor
DB03147	0.513	0.398	0.510	0.208	0.161	0.204	FAD	Vitamin
DB05351	0.771	0.596	0.773	0.473	0.360	0.473	Dexlansoprazole	Proton Pump inhibitor
DB00448	0.771	0.596	0.773	0.473	0.360	0.473	Lansoprazole	Proton Pump inhibitor
DB12808	0.617	0.478	0.615	0.281	0.246	0.279	Trifarotene	Retinoid Cream
DB00678	0.869	0.735	0.871	0.668	0.512	0.674	Losartan	ACE2 receptor blocker
DB00605	0.748	0.570	0.750	0.453	0.352	0.453	Sulindac	COX2 inhibitor
DB11641	0.608	0.494	0.605	0.362	0.286	0.357	Vinflunine	Tubulin: Anti-tumour
DB01026	0.693	0.534	0.693	0.405	0.327	0.404	Ketoconazole	Antifungal
DB06787	0.680	0.529	0.679	0.401	0.330	0.400	Hexocyclium	Muscarinic: replace by PPI's

SSnet scores are probability, in the range from 0 to 1, of a drug binding with a IC50 less then 10nM. Smina values are provided in kcal/mol. Values represent mean of 3 replicas. The deviation reported is calculated using the average of absolute deviations from the mean values of the 3 smina replicas.

Top Scores for SSnet ACE2 (open) - ACE2:S1 (closed) with Zinc.

Table S6 shows the top compounds ranked according SSnet score in presence of Zinc.

Table S6: Top Scores for SSnet ACE2 (open) - ACE2:S1 (closed) with Zinc.

Drug name	smina ACE2 (open)	smina ACE2:S1 (closed)	smina ACE2 (closed)	SSnet ACE2 (open)	SSnet ACE2:S1 (closed)	SSnet ACE2 (closed)	Common name	Usage	Mode of Action
DB13125	-7.00 ±0.20	-7.60 ±0.73	-7.23 ±0.98	0.712	0.500	0.713	Lusutrombopag	Treatment of thrombocytopenia	Thrombopoietin receptor (TPOR) agonist
ZINC000100054334	-8.37 ±0.11	-8.17 ±0.11	-8.30 ±0.20	0.677	0.466	0.678	P-O-Glucuronide Rosiglitazone	Metabolite of rosiglitazone	Not applicable
ZINC000095618628	-8.33 ±0.24	-8.47 ±0.22	-8.27 ±0.36	0.668	0.458	0.668	3-Hydroxynevirapine Glucuronide	Nevirapine metabolite (HIV treatment)	Reverse transcriptase inhibitor
ZINC000013682481	-7.87 ±0.24	-8.00 ±0.13	-7.73 ±0.31	0.641	0.432	0.641	Elvitegravir	Treatment of HIV	Integrase inhibitor
DB08966	-6.43 ±0.09	-6.60 ±0.07	-6.57 ±0.09	0.638	0.429	0.639	Fursultiamine	Thiamine deficiency	Disulfide derivative of thiamine
ZINC000040165220	-10.00 ±0.27	-9.93 ±0.51	-10.30 ±0.27	0.652	0.447	0.652	Estriol-3-glucuronide	Human metabolite of 16α, 17β-estriol	Not applicable
ZINC000014089740	-9.43 ±0.16	-9.63 ±0.04	-9.53 ±0.11	0.693	0.488	0.695	Morphine-6-Glucuronide	Active metabolite of morphine	Opiod receptor binder
DB04570	-8.33 ±0.16	-8.47 ±0.04	-8.37 ±0.04	0.662	0.460	0.664	Latamoxef	Antibiotic (Second-Generation Cephalosporin)	Inibition of cross-linking of the peptidoglycan
DB00683	-7.93 ±0.58	-8.13 ±0.44	-7.73 ±0.71	0.704	0.502	0.705	Midazolam	Sedative	Enhances inhibitory action of GABA
ZINC000002516030	-8.06 ±0.11	-8.13 ±0.04	-8.00 ±0.13	0.724	0.524	0.724	Hydroxy haloperidol	Schizofrenia	Not applicable
ZINC000008215517	-7.30 ±0.33	-7.57 ±0.18	-7.17 ±0.42	0.691	0.491	0.693	Cocarboxylase	Thiamine derivative	Biochemical catalysts
ZINC000004175630	-9.17 ±0.09	-9.50 ±0.27	-9.37 ±0.36	0.692	0.492	0.691	Orap	Antipsychotic agent	Possibly acting on postsynaptic dopamine receptors

SSnet scores are probability, in the range from 0 to 1, of a drug binding with a IC50 less then 10nM. Smina values are provided in kcal/mol. Values represent mean of 3 replicas. The deviation reported is calculated using the average of absolute deviations from the mean values of the 3 smina replicas.

Smina and SSnet scores for top scoring compounds according to smina without restriction on number of atoms

Table S7 shows the top scoring compounds ranked on the basis of smina score when no cutoff on the number of atoms is applied. It can be noted that the top binding compounds present a large deviation associated with their smina energies. The deviation reported is calculated using the average of absolute deviations from the mean values of the 3 smina replicas. A significant amount of the compounds in this table are macrolides with an antibiotic function.

Table S7: Smina and SSnet scores for top scoring compounds according to smina without restriction on number of atoms.

**Drug name**		**smina**			**SSnet**		**Common name**	**Class**	**Usage**
	**ACE2 (open)**	**ACE2:S1 (closed)**	**ACE2 (closed)**	**ACE2 (open)**	**ACE2:S1 (closed)**	**ACE2 (closed)**			
NuBBE_1255	-13.43 +/- 0.56	-14.30 +/- 0.80	-13.57 +/- 1.29	0.025	0.029	0.024	β-Pedunculagin	Hydrolysable Tannin	Anti-inflammatory, anticancer, antioxidant.
NuBBE_1179	-13.53 +/- 0.24	-13.90 +/- 0.00	-13.57 +/- 0.22	0.016	0.019	0.016	castalagin	Hydrolysable Tannin	Antioxidant, antitumorigenic
NuBBE_1178	-13.43 +/- 0.24	-13.83 +/- 0.71	-13.57 +/- 0.89	0.020	0.024	0.020	casuarinin	Hydrolysable Tannin	Antitrypanosomal, Antileishmanial
DB00615	-12.43 +/- 1.76	-13.20 +/- 2.00	-13.30 +/- 1.93	0.928	0.884	0.929	Rifabutin	Macrolactams	Broad-spectrum antibiotic
DB01201	-11.87 +/- 1.84	-13.30 +/- 1.53	-12.93 +/- 2.09	0.980	0.966	0.981	Rifapentine	Macrolactams	Anti tubercolosis antibiotic
DB01045	-11.70 +/- 1.27	-12.90 +/- 1.80	-12.67 +/- 1.96	0.971	0.950	0.971	Rifampicin	Macrolactams	Broad-spectrum antibiotic
DB00644	-12.80 +/- 0.3	-12.10 +/- 1.20	-11.97 +/- 1.11	0.925	0.853	0.928	Gonadorelin	Gonadotropin-releasing hormone (peptide)	Ovulation induction therapy
SANC00526	-11.70 +/- 0.93	-12.23 +/- 0.49	-12.57 +/- 0.16	0.000	0.001	0.000	1',2-Binaphthalen-4-one-2',3-dimethyl-1,8'-epoxy-1,4',5,5',8,8'-hexahydroxy-5',8-di-O-β-xylopyranosyl ($1\rightarrow6$)-β-glucopyranoside	Binaphthalenone	Antibacterial
NuBBE_1967	-11.73 +/- 0.64	-12.40 +/- 0.40	-11.40 +/- 0.27	0.088	0.102	0.086	Gnetin-E	Phenylpropanoids	None associated
NuBBE_200	-11.63 +/- 0.96	-12.27 +/- 0.49	-11.17 +/- 1.22	0.054	0.064	0.053	Amentoflavone	Flavonoids	Topical anti-inflammatory
ZINC000169621200	-11.57 +/- 0.11	-11.87 +/- 0.42	-11.97 +/- 0.35	0.965	0.934	0.966	Rifaximin	Macrolactams	Antibiotic (E. coli)
SANC00491	-9.60 +/- 0.00	-11.80 +/- 0.00	-11.20 +/- 0.00	0.879	0.791	0.880	Cephalostatin 17	Bis-steroidal	Potential anti-leukimia treatment
DB00007	-11.00 +/- 0.27	-11.23 +/- 0.58	-11.80 +/- 0.67	0.990	0.979	0.991	Leuprolide	Synthetic peptide hormone	Prostate cancer, uterine fibroids, and endometriosis.
ZINC000164528615	-10.83 +/- 0.44	-11.13 +/- 0.78	-11.77 +/- 0.36	0.998	0.995	0.998	Glecaprevir	Antiviral	Hepatitis C
NuBBE_288	-11.00 +/- 0.60	-11.77 +/- 0.18	-10.70 +/- 0.53	0.006	0.007	0.005	Procyanidin B-3	Flavonoids	Antioxidant
SANC00524	-11.50 +/- 0.53	-11.73 +/- 0.58	-11.63 +/- 0.64	0.001	0.001	0.000	1',2-Binaphthalen-4-one-2',3-dimethyl-1,8'-epoxy-1,4',5,5',8,8'-hexahydroxy-8-O-β-glucopyranosyl-5'-O-β-xylopyranosyl ($1\rightarrow6$)-β-glucopyranoside	Binaphthalenone	Antimicrobial
SANC00559	-11.47 +/- 0.38	-11.70 +/- 0.13	-11.23 +/- 0.44	0.012	0.014	0.011	Epimesquitol-($4\beta\rightarrow5$)-3,3',4',7,8-pentahydroxy flavonone	Flavonoid	None associated
SANC00479	-9.10 +/- 0.00	-11.70 +/- 0.00	-10.50 +/- 0.00	0.799	0.700	0.798	Cephalostatin 3	Alkaloid, steroid, cephalostatin	Anticancer
SANC00558	-11.30 +/- 0.40	-11.67 +/- 0.36	-11.17 +/- 0.69	0.012	0.014	0.011	Epimesquitol-($4\beta\rightarrow5$) -3,3',4',7,8-pentahydroxy flavonone	Flavonoid, Promelacacinidin	None associated
DB00512	-11.20 +/- 0.73	-11.67 +/- 0.71	-11.07 +/- 0.31	0.993	0.987	0.993	Vancomycin	Glycopeptide related	Antibacterial (Staphylococcus aureus)

SSnet scores are probability, in the range from 0 to 1, of a drug binding with a IC50 less then 10nM. Smina values are provided in kcal/mol. Values represent mean of 3 replicas. The deviation reported is calculated using the average of absolute deviations from the mean values of the 3 smina replicas.

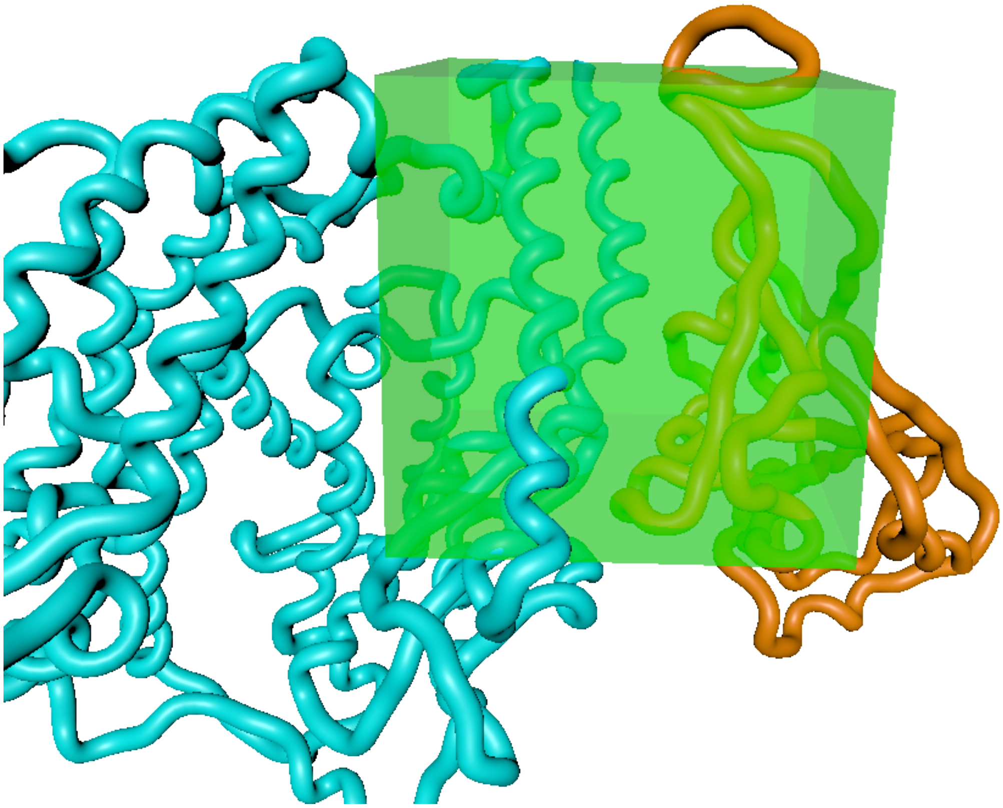

Figure S1: ACE2:S1 complex with box used for docking. In blue ACE2 receptor. In orange S1 sub-unit of the spike protein. In green box used for the smina docking.

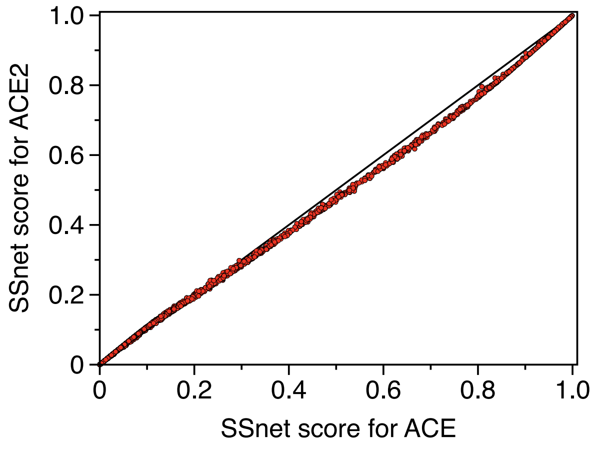

(a) 6M17

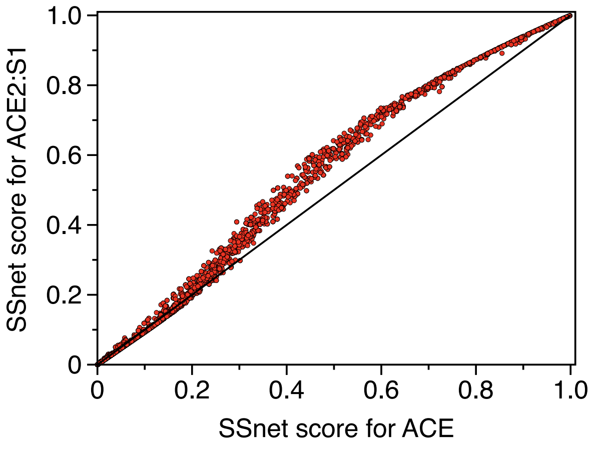

(b) 6M1D

Figure S2: SSnet scores on ACE and ACE2

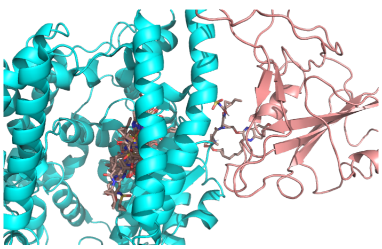
(a) ZINC000095551509

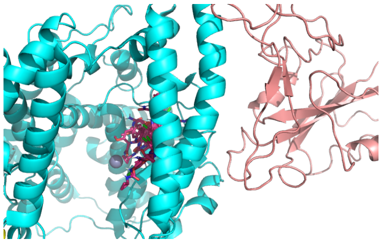
(b) DB09078

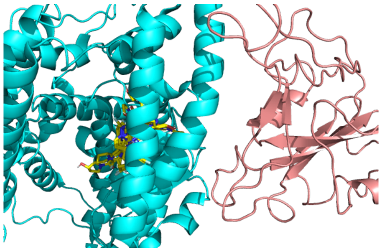
(c) DB14723

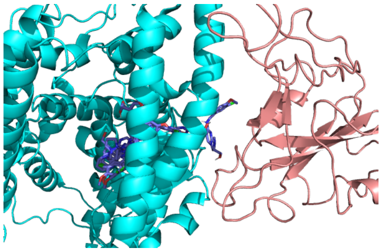
(d) ZINC000011677857

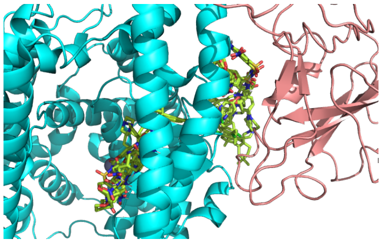
(e) DB11581

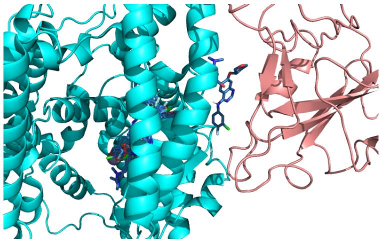
(f) DB08916

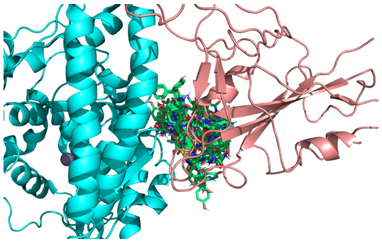
(g) DB08890

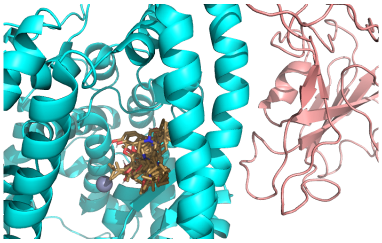
(h) ZINC000001319780

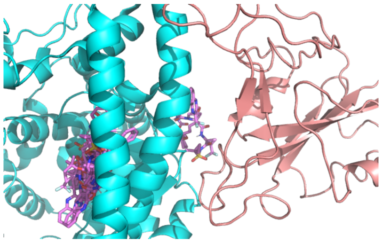
(i) DB13879

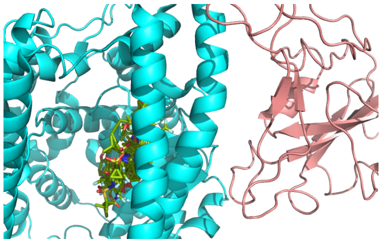
(j) ZINC000218037687

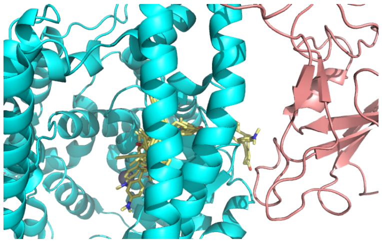
(k) DB00834

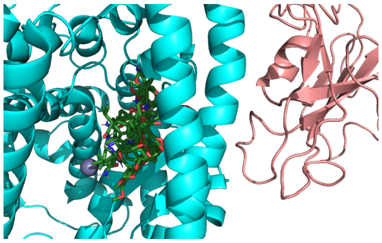
(l) ZINC000004393164

Figure S3: Binding poses for the compounds listed in Table S2

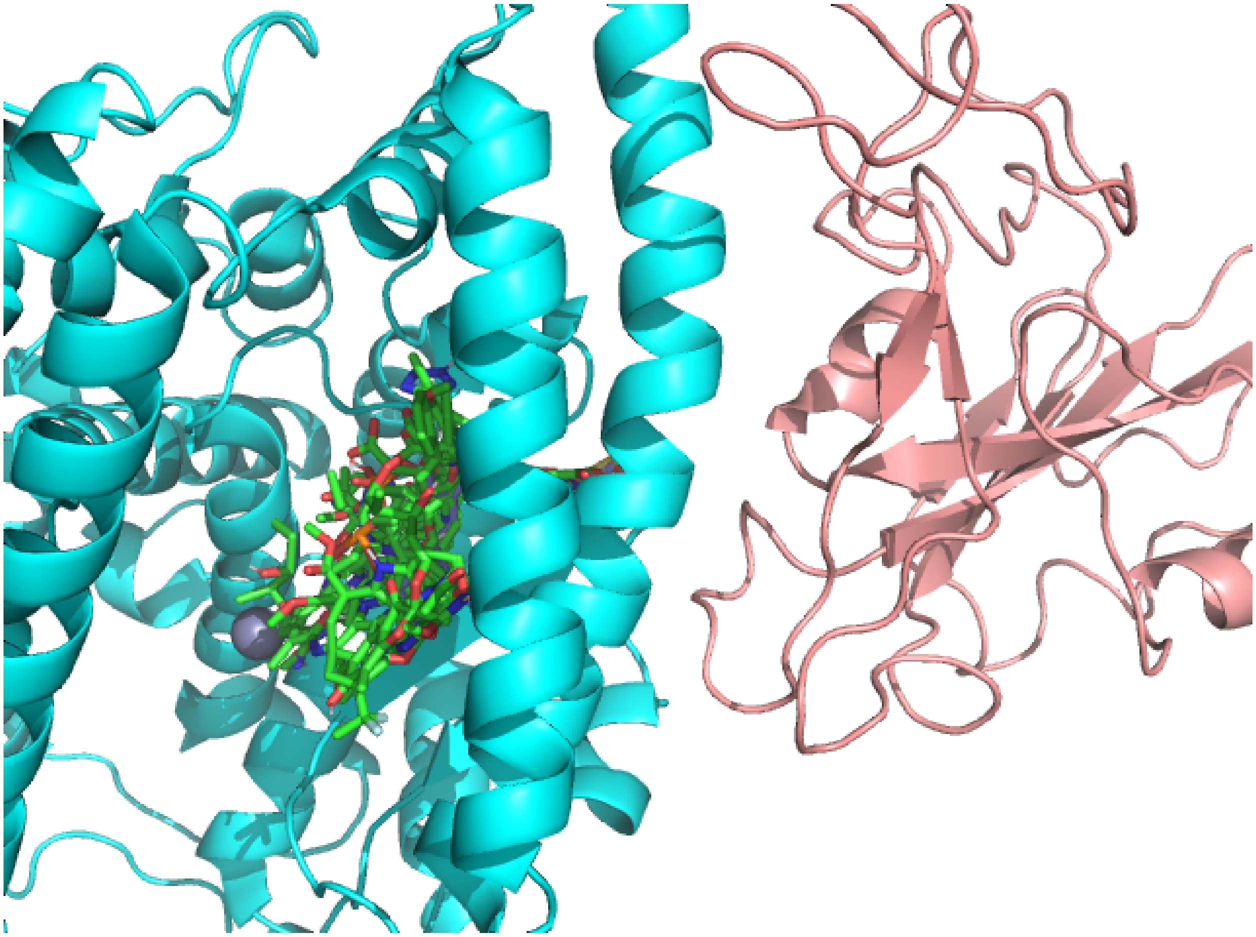

Figure S4: Top 15 ligands that are significantly affected by considering Zn in SSnet.

Defective 3D structures

The following list of DrugBank, ZINC, NuBBE and SANC IDs are the compounds with incorrect 3D structures when converting from SMILES.

DB14702	ZINC000100054873	ZINC000169987861
DB11590	ZINC000261494693	ZINC000261494596
ZINC000100003506	ZINC000261527167	ZINC000261494599
ZINC000100003201	ZINC000100053566	ZINC000100054182
ZINC000100003504	ZINC000100353857	ZINC000261494598
ZINC000100005085	DB00462	ZINC000100054187
ZINC000100036955	ZINC000005497532	ZINC000003915154
ZINC000100036957	ZINC000100053083	ZINC000014261579
ZINC000100003200	ZINC000100053563	ZINC000118913630
ZINC000100052205	ZINC000100068871	ZINC000118915338
ZINC000100002769	ZINC000100353864	ZINC000040164488
ZINC000100022260	DB00757	ZINC000118915336
ZINC000100002768	ZINC000004215173	ZINC000118915339
ZINC000100022257	ZINC000015449189	ZINC000257459143
ZINC000022053867	ZINC000100036938	ZINC000118915337
ZINC000022053870	ZINC000100053074	ZINC000257526034
DB05389	ZINC000005167162	DB00320
ZINC000212549525	ZINC000100054893	ZINC000118915331
ZINC000100037714	ZINC000257384247	ZINC000256315101
ZINC000212549609	ZINC000101144764	ZINC000095617624
ZINC000100037710	ZINC000257384244	ZINC000256315095
ZINC000100053860	ZINC000261494628	ZINC000256315111
ZINC000150307441	ZINC000100054890	ZINC000118913648

ZINC000100053849	ZINC000242723020	DB09297
ZINC000100053855	DB12161	ZINC000256315106
ZINC000009411213	ZINC000100037885	ZINC000118913649
ZINC000100003677	ZINC000100037890	ZINC000169621209
ZINC000100035525	ZINC000261494622	ZINC000169621210
ZINC000100042145	DB09015	ZINC000256641128
ZINC000100053845	ZINC000100017742	ZINC000256640979
ZINC000100042076	DB00995	ZINC000085552271
ZINC000100003676	ZINC000012494340	ZINC000100053092
ZINC000002008391	ZINC000256479159	ZINC000299818014
ZINC000100054361	ZINC000005167145	ZINC000118915215
ZINC000100390602	ZINC000100017736	ZINC000085537011
ZINC000000402954	ZINC000003876023	ZINC000095862733
ZINC000002008394	ZINC000004097304	ZINC000299872475
ZINC000100054365	ZINC000095618838	ZINC000096006012
ZINC000012503137	ZINC000095889730	ZINC000299872477
ZINC000100051396	ZINC000103561250	ZINC000299872478
ZINC000104344008	ZINC000261515676	ZINC000299872476
ZINC000100003507	DB00860	DB13995
ZINC000100003509	ZINC000008220548	ZINC000261494615
ZINC000100051395	ZINC000169621213	ZINC000255962815
ZINC000100053185	ZINC000169621217	ZINC000245190610
ZINC000100054812	ZINC000251257402	DB00115
ZINC000009212324	ZINC000253387331	DB09385
ZINC000100051302	DB00957	DB00200
ZINC000100053305	ZINC000106400715	DB06402
ZINC000169339296	ZINC000169621214	DB11191

ZINC000005513515	ZINC000082004496	NuBBE_1669
ZINC000100053191	DB00253	NuBBE_2010
ZINC000100053295	DB14583	NuBBE_1551
ZINC000100053310	DB14646	NuBBE_1360
ZINC000100054130	ZINC000044963475	NuBBE_1786
ZINC000100054813	ZINC000090716806	NuBBE_1552
ZINC000221408371	ZINC000005459685	NuBBE_429
ZINC000261494583	ZINC000118913726	NuBBE_1366
ZINC000003831582	ZINC000261494643	NuBBE_2520
ZINC000003871891	ZINC000005459684	NuBBE_2301
ZINC000083294652	ZINC000100054235	NuBBE_1530
ZINC000100053300	ZINC000230122970	NuBBE_2152
ZINC000100054124	DB01234	NuBBE_2149
ZINC000100054840	ZINC000008679996	NuBBE_1935
ZINC000256421827	DB14540	NuBBE_1705
ZINC000261494582	ZINC000004213353	NuBBE_2148
ZINC000261494603	ZINC000261494664	NuBBE_1228
ZINC000003872494	ZINC000004097440	NuBBE_1548
ZINC000100018598	ZINC000084441937	NuBBE_997
ZINC000261494670	ZINC000169621195	NuBBE_2017
DB00956	ZINC000100013130	NuBBE_1675
ZINC000003872493	ZINC000100036924	NuBBE_1005
ZINC000100051205	ZINC000100038287	NuBBE_2235
ZINC000100054856	ZINC000169621196	NuBBE_2407
ZINC000110344463	DB00596	NuBBE_1576
ZINC000085552393	ZINC000100038281	NuBBE_1578
ZINC000100018594	ZINC000238730561	NuBBE_2409

ZINC000100054849	DB14669	NuBBE_1354
ZINC000118912393	ZINC000004097439	NuBBE_1483
ZINC000253497554	ZINC000038611810	NuBBE_440
ZINC000230122947	ZINC000103592099	NuBBE_989
ZINC000004428529	ZINC000238730560	NuBBE_1514
ZINC000100027531	ZINC000004212626	NuBBE_1044
ZINC000100054081	ZINC000238730523	NuBBE_682
ZINC000100054225	ZINC000003995807	NuBBE_2234
ZINC000256335049	ZINC000095626782	NuBBE_381
DB09371	ZINC000118915223	NuBBE_1052
ZINC000100036756	ZINC000256296829	NuBBE_1719
ZINC000100054077	ZINC000004099035	NuBBE_189
ZINC000100054084	ZINC000014880004	NuBBE_1034
ZINC000101210593	ZINC000118915222	NuBBE_72
ZINC000256335053	ZINC000100073786	NuBBE_1033
DB00977	ZINC000100370145	NuBBE_1163
ZINC000100014791	ZINC000111460375	NuBBE_2193
ZINC000100053366	DB06777	NuBBE_1035
ZINC000100054079	ZINC000095618776	NuBBE_316
ZINC000101210596	ZINC000100054219	SANC00582
ZINC000100057533	ZINC000003977777	SANC00581
ZINC000100054871	ZINC000261494597	SANC00140
SANC00133	SANC00551	SANC00218
SANC00518	SANC00141	SANC00552
